# P-2093. A 30-year Descriptive Analysis of *Mycobacterium szulgai* Infections

**DOI:** 10.1093/ofid/ofae631.2249

**Published:** 2025-01-29

**Authors:** Pool Tobar Vega, Holenarasipur R Vikram, Alyssa McGary

**Affiliations:** Mayo Clinic School of Science, Pensacola, Florida; Mayo Clinic, Phoenix, Arizona; Mayo Clinic School of Science, Pensacola, Florida

## Abstract

**Background:**

*Mycobacteirum Szulgai* is a non-tuberculous mycobacterium known to cause human disease. Descriptions in the literature are limited to case reports and a larger case series from the Netherlands. Guidelines from the American Thoracic Society recommend that M. szulgai should always be considered pathogenic and that treatment should be based on in-vitro susceptibilities.

Demographics and patient characteristics

**Figure:**
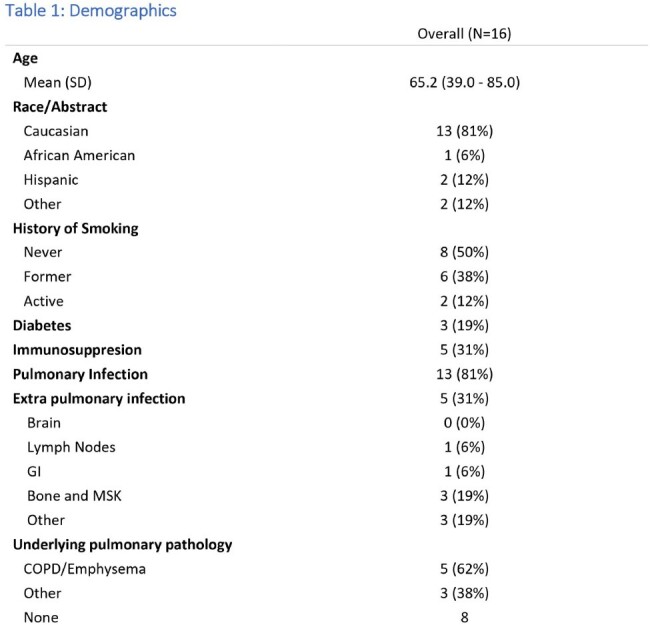

**Methods:**

We embarked on a retrospective study of adults ( >18 years) with *M. szulgai* infection at the Mayo Clinic sites in Arizona, Minnesota, and Florida. We queried the Mayo Clinic Database using two different search methods, (Advanced Text Explorer and the Mayo Data Explorer) using the terms ‘Mycobacterium Szulgai,’ ‘Mycobacteria Szulgai’,’ M. Szulgai’ and ‘Szulgai.’ Medical records from 1994 to 2023 were reviewed.

Diagnostic studies

**Figure:**
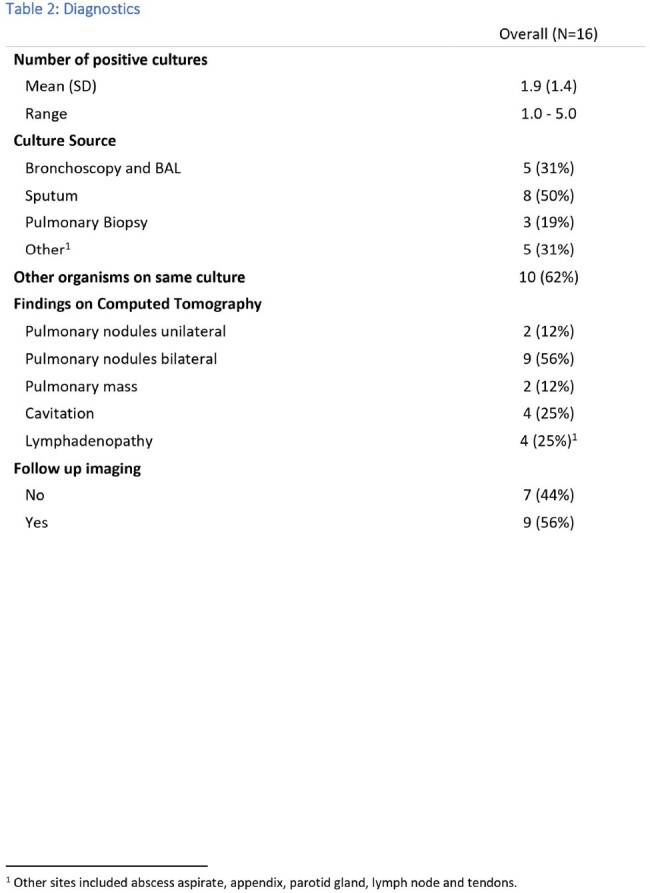

**Results:**

Initial search yielded 43 patients. Twenty-seven patients were excluded (incomplete records or prior history of infection treated elsewhere). For our cohort of 16 patients, the mean age was 65 years [IRQ 57-74 years], 13 of those were caucasian. Five patients [31%] were immunosuppressed. Thirteen patients [81%] developed pulmonary infection, 5[31%] had extra pulmonary infection (2[12%] with disseminated disease and 3[19%] with extra-pulmonary disease without primary pulmonary infection). Only 8 [50%] patients received specific therapy. All treated patients met ATS criteria for NTM infections, while 3 [19%] untreated patient met criteria. Only 4 [25%] patient received therapy based on susceptibilities. The most common regimen was a combination of rifampin, ethambutol and macrolide in 5[31%] of the patients. Seven patients [47%] were cured, 4[27%] had ongoing symptoms, 1[7%] had relapse and 1 [7%] is currently receiving therapy. There were 2[ 13%] deaths in the untreated group and none from the treated group. One patient was lost to follow up.

Treatment and outcomes

**Figure:**
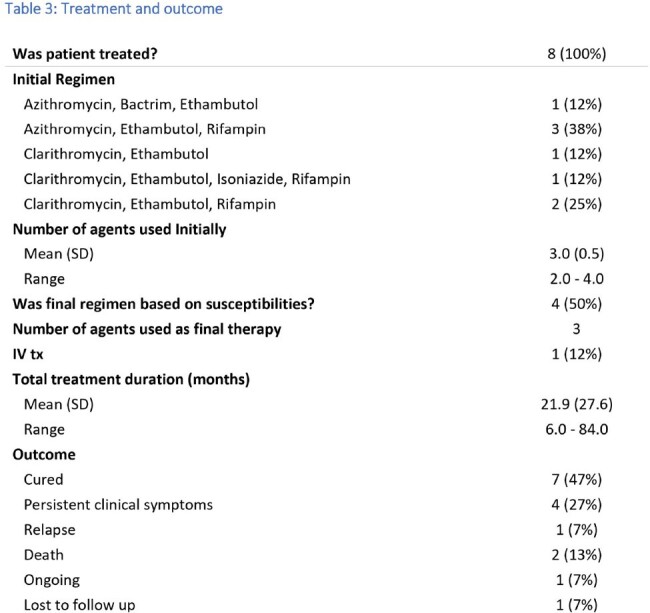

**Conclusion:**

Our findings echo published literature and ATS recommendations that *M. szulgai* should be regarded as a true pathogen that necessitates targeted therapy.

**Disclosures:**

All Authors: No reported disclosures

